# Large river habitat complexity and productivity of Puget Sound Chinook salmon

**DOI:** 10.1371/journal.pone.0205127

**Published:** 2018-11-01

**Authors:** Jason E. Hall, Correigh M. Greene, Oleksandr Stefankiv, Joseph H. Anderson, Britta Timpane-Padgham, Timothy J. Beechie, George R. Pess

**Affiliations:** 1 Fish Ecology Division, Northwest Fisheries Science Center, National Marine Fisheries Service, National Oceanic and Atmospheric Administration, Seattle, WA, United States of America; 2 Ocean Associates, Inc., Under contract to Northwest Fisheries Science Center, National Marine Fisheries Service, National Oceanic and Atmospheric Administration, Seattle, WA, United States of America; 3 Washington Department of Fish and Wildlife, Olympia, WA, United States of America; Texas A&M University, UNITED STATES

## Abstract

While numerous studies have shown that floodplain habitat complexity can be important to fish ecology, few quantify how watershed-scale complexity influences productivity. This scale mismatch complicates population conservation and recovery strategies that evaluate recovery at regional or multi-basin scales. We used outputs from a habitat status and trends monitoring program for ten of Puget Sound’s large river systems to examine whether juvenile Chinook salmon productivity relates to watershed-scale habitat complexity. We derived habitat complexity metrics that quantified wood jam densities, side and braid to main channel ratios, and node densities from a remote sensing census of Puget Sound’s large river systems. Principal component analysis revealed that 91% of variance in these metrics could be explained by two principal components. These metrics revealed gradients in habitat complexity across Puget Sound which were sensitive to changes in complexity as a result of restoration actions in one watershed. Mixed effects models revealed that the second principle component term (PC2) describing habitat complexity was positively related to log transformed subyearling Chinook per spawner productivity rates from 6–18 cohorts per watershed. Total subyearling productivity (subyearlings per spawner) and fry productivity (subyearling fry per spawner) rates were best described by models that included a positive effect of habitat complexity (PC2) and negative relationships with log transformed peak flow recurrence interval, suggestive of reduced survival due to egg destruction during floods. Total subyearling productivity (subyearlings per spawner) and parr productivity (subyearling parr per spawner) rates were best described by models that included a positive effect of habitat complexity (PC2) and negative relationships with log transformed spawner density, suggestive of density dependent limits on juvenile rearing habitat. We also found that coefficient of variation for log transformed subyearling productivity and subyearling fry productivity rates declined with increasing habitat complexity, supporting the idea that habitat complexity buffers populations from annual variation in environmental conditions. Therefore, we conclude that our watershed-scale census-based approach provided habitat complexity metrics that explained some of the variability in productivity of subyearling juveniles among Chinook salmon populations. Furthermore, this approach may provide a useful means to track and evaluate aggregate effects of habitat changes on the productivity of Endangered Species Act (ESA) listed Chinook salmon populations over time.

## Introduction

The idea that habitat complexity, as described by the diversity of different habitat types or features within an area, offers benefits to mobile animals is central to conservation biology, and many studies have identified the importance of habitat heterogeneity or complexity for multispecies conservation [1]. While the idea that habitat complexity supports community complexity has long been central to ecology [2], less attention has focused on how habitat complexity affects demography in individual species or populations. From community ecology, much research on demographic benefits of habitat complexity focuses on reduced inter- or intraspecific interactions such as competition [3–4] or predation [5–6]. More broadly, researchers have postulated that spatial complexity provides variance buffering [7]. Here we examine whether productivity rates from multiple populations of an anadromous fish track measurements of habitat complexity in freshwater rearing habitats at large spatial scales.

Freshwater habitat complexity and connectivity are key features for aquatic species [8,9,10] and affect individual condition, behavior, and survival [11–13]. The amount and arrangement of habitat has individual and population scale impacts [14–15]. Although examples illustrating connections between habitat complexity and demographic rates exist, most are short-term (e.g., ≤ several years) or spatially restricted (e.g., reaches or one watershed) [12,16–17]. Efforts relating habitat complexity to occupancy, abundance, and productivity at larger spatial (e.g., regional) and temporal extents (e.g., ≥10 years) have grown recently [15,18–20]. Larger spatial and temporal scale analyses have also revealed how environmental variables like river discharge or temperatures affect fish populations [10,21–22]. Thus, consistent time-series of large-scale habitat data paired with demographic data can support management and recovery efforts by identifying important habitat relationships to monitor.

Floodplain habitats remain important to fish populations despite widespread losses of habitat complexity (e.g., channel straightening and confinement that tend to convert floodplain rivers to single thread channel systems) and floodplain connectivity (e.g., diking and levees to prevent flooding onto floodplain surfaces and into floodplain habitats) from anthropogenic modifications [23–25]. Floodplains offer additional rearing capacity for fishes, especially during the wet months when juvenile fish seek rearing opportunities in floodplain habitats that facilitate increased growth and survival by offering abundant prey, optimal rearing temperatures, and refuge from predators [12,16–17,26]. Perhaps most critically for juvenile fishes, floodplains offer protection from floods by attenuating high flows and providing refuge habitats from high flow conditions in the main channel network [12,27]. Given widespread losses and the potential benefits of floodplain habitats, scientists are increasingly recognizing the importance of restoring habitat complexity and floodplain connectivity to recovery of threatened species [23–24]. However, what constitutes “complex” floodplain habitat and how the cumulative benefits of habitat complexity affect productivity at population scales remains largely unknown. Furthermore, consistent monitoring of habitat complexity is not typically available at the spatial or temporal scales needed to manage fish populations, which are typically managed at watershed and regional scales as opposed to within individual habitats or reaches that are often the focus of research and monitoring efforts.

In this paper, we address this knowledge gap by taking advantage of a large-scale habitat census initiated within the Puget Sound region of Washington State, USA [28]. This region supports all Pacific salmon species, including populations of Chinook salmon (*Oncorhynchus tshawytscha*) listed as Threatened under the Endangered Species Act (ESA). These anadromous salmon rely on large river habitats at several life stages, with adults spawning in Puget Sound’s large rivers, eggs incubating in river substrates for several months, juveniles rearing in river habitats for days to a year, and juveniles migrating to the ocean to mature before returning back to the river as an adult to spawn. Although long-term data describing the abundance of juvenile salmon migrating to the ocean and the number of adults returning to spawn in Puget Sound’s large river systems currently exist, complimentary and consistent habitat data within Puget Sound measured at the watershed scale have been lacking. To address this need, we used a census-based remote sensing approach to quantify main, side, and braid channel features as well as wood jams throughout Puget Sound’s large river systems to develop consistent, watershed scale measures of large river habitat quantity and complexity.

We hypothesized that this census-based remote sensing approach would capture differences in habitat quantity and complexity, among large river systems in the Puget Sound region. Furthermore, we hypothesized that our approach would detect watershed-scale changes in large river habitat quantity and complexity as a result of habitat change over time (e.g., restoration and natural change). If watershed-scale habitat complexity increases the productivity of fish populations, we hypothesized that aggregate fish productivity measures at the watershed scale would be positively related to watershed-scale measurements of habitat complexity. Furthermore, we hypothesized that if habitat complexity buffers annual variability in the productivity of these watersheds, measures of variation in annual productivity would be negatively related to watershed-scale habitat complexity. To evaluate relationships between habitat complexity and the productivity rates of juvenile Chinook salmon, we used an information-theoretic model selection approach to evaluate regional relationships between Chinook population freshwater productivity and basin-scale habitat complexity.

## Materials and methods

### Study area

**Fig 1:**
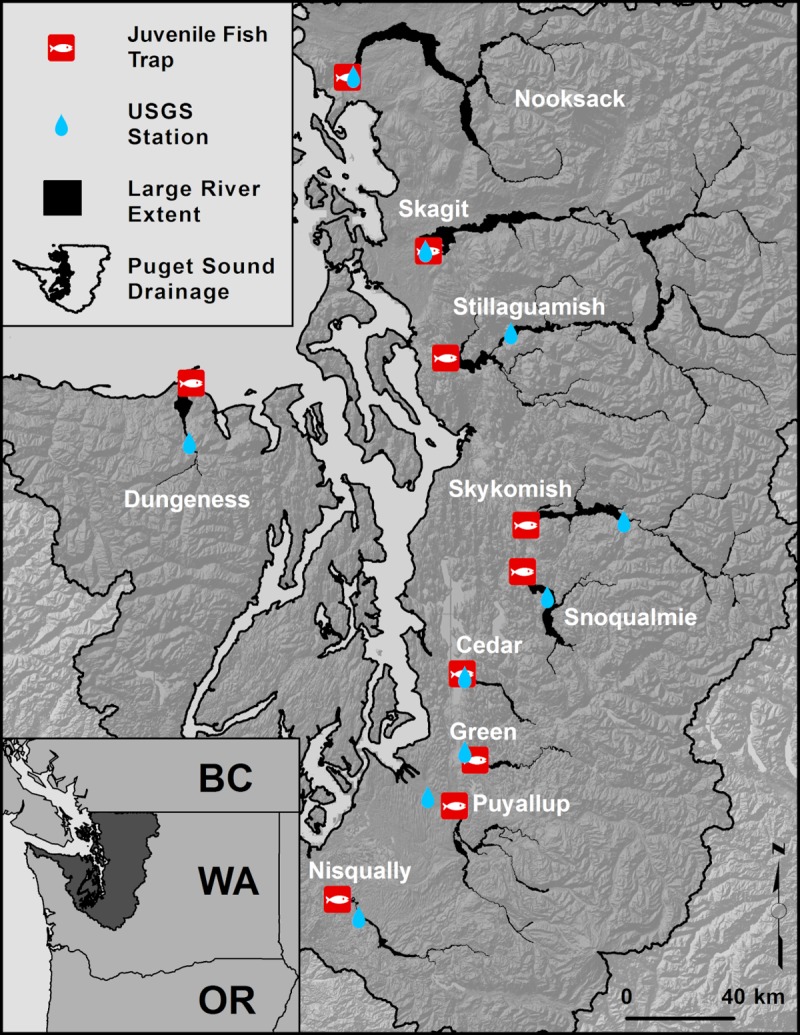
Study area map of large river systems in Puget Sound. Puget Sound drainage located in Washington State of the northwestern United States (see inset) and the extent of large river habitat mapping, location of smolt traps, and location of USGS river flow stations used in the analysis. USGS stations shown include 12200500, 12113000, 12048000, 12089500, 12119000, 12134500, 12149000, 12167000, 12213100, and 12101500.

The analysis presented here focuses on ten large river watersheds that drain into the Puget Sound located in the Pacific Nonwestern United States in the State of Washington (Fig 1). From the northern extent of Puget Sound near the Canadian board to Puget Sound’s southern most extent near the capital city of Olympia in Washington State, we considered the Nooksack, Skagit, Stillaguamish, Skykomish, Snoqualmie, Cedar, Green, Puyallup, and Nisqually Rivers (Fig 1). We also considered the Dungeness River which drains into the Strait of Juan de Fuca connecting the Puget Sound to the northeastern Pacific Ocean (Fig 1). These watersheds represent a gradient of geomorphic settings, with mountain valleys and lowland valleys being formed by continental glaciers, alpine glaciers, and subsequent incision and deposition of sediments from rivers. Within this setting, these rivers occur in a temperate climate with elevation gradients that result in a gradient of hydrological settings from rainfall-dominated (<790 m elevation with peak river discharges in December–February) to snowmelt-dominated (>1300 m elevation with peak river discharges in May–June) watersheds [21]. In addition, land use histories and development have resulted in gradients of industrial and urbanization in Puget Sound’s central valleys to more agricultural development in the northern and southern extents of Puget Sound [28]. These variations in geomorphic, hydrologic, and land use patterns, combined with the presence of ESA listed Chinook salmon populations and the availability of long-term population monitoring programs, presented a unique setting to examine the relationship between large river habitat complexity and population scale productivity of fish populations.

Within the Puget Sound watersheds that we considered in this analysis, we further restricted our analysis extent to areas upstream of juvenile fish traps that are operated by Washington Department of Fish and Wildlife (WDFW), Tulalip Tribes, Stillaguamish Tribe of Indians, Puyallup Tribe of Indians, and Lummi Nation. The upstream extent of our analysis was limited by a minimum upstream drainage area of 50 km^2^ and at known barriers to migrating adult salmon that migrate upstream each year to spawn in these watersheds. These barriers include natural high gradient barriers (e.g., water falls) and anthropogenic barriers (e.g., dams) that limit the upstream extent of adult Chinook migrations [28].

### Habitat complexity

**Fig 2:**
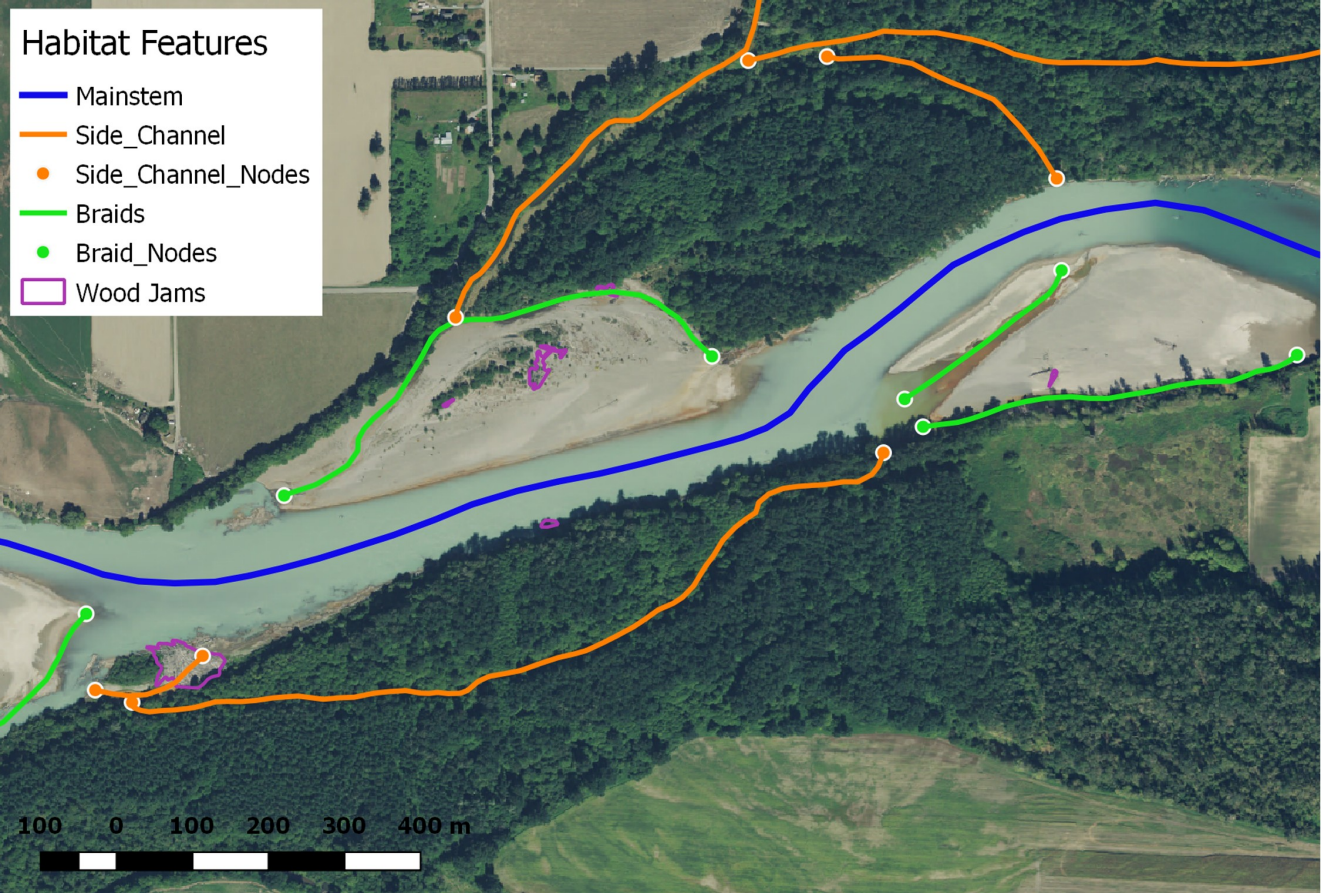
Illustration of digitized habitat features. Example of digitized habitat features in a representative segment of the Skagit River within our study area. The actual aerial image shown here is for illustrative purposes and is representative of the 2015 Google aerial imagery used in our analysis. The aerial image shown here is a 2015 National Agriculture Imagery Program (NAIP) image obtained through the United States Geological Survey (USGS) National Map Viewer (http://viewer.nationalmap.gov/viewer/). Note that braids and side channels are digitized to the edge of the main channel rather than the centerline, which provides a more accurate and repeatable length measurement. Note that the number of nodes equals twice the number of braid and side channel features.

Within the study area (e.g., above juvenile fish traps, below adult Chinook migration barriers, and below where drainage area was greater than 50 km^2^ for all ten watersheds, Fig 1), we digitized large river habitat features in the WGS 84 Pseudo Mercator (EPSG:3857) coordinate reference system from 0.3-meter Google images flown in 2013–2016 during summer leaf-on conditions in ArcMap GIS Software (Version 10.3). We digitized all main, braid, and side channels; nodes of channel bifurcations and confluences; and wood jams within the study area (Fig 2 and Table 1). These feature types were selected because they could be consistently derived from readily available aerial photography with low signal to noise ratios and observer errors, and remote sensing could be used to obtain a census measurement of these habitat features more efficiently than costlier field-based methods [29,30]. From these features, we derived five metrics to describe habitat complexity for each watershed within our study area. These habitat complexity metrics included side and braid channel length ratios (km side or braid channel/km main channel), side and braid channel node densities (side or braid nodes/km main channel), and wood jam densities (m^2^ wood jam/km main channel) (Table 1). These metrics have been shown to be related to salmon population metrics (e.g., abundance, productivity, and growth) at different scales (e.g., within and between rivers), and these metrics are sensitive to land management and restoration actions [29,31–35]. As noted in Table 1, all metrics were adjusted by mainstem channel length to account for differences in watershed size. The digitized large river habitat features and resulting habitat quantity and complexity metrics for each of the ten watersheds in our study area represent a snap shot of large river habitat based on the range of aerial photograph acquisition dates used in this study (circa 2013–2016). To evaluate the ability of our approach to detect changes in habitat over time, we also digitized features from archived aerial imagery (acquired in 2009 and 2012 during summer leaf-on conditions) within one watershed (Cedar River, Fig 1) where two restoration projects created large river habitat features [36–37] that could be used to quantify pre- and post-restoration habitat quantity and complexity.

**Table 1:** Habitat complexity metrics.

Habitat complexity metric	Description
Braid: main channel length ratio	Ratio of braid length to main channel length (km/km). Braids are secondary flow paths separated from the main flow path by unvegetated gravel bars.
Braid node density	Number of junctions between braids and the main channel, side channels, or other braids per kilometer of main channel. Number of nodes therefore equals two times the number of braids.
Side channel: main channel length ratio	Ratio of side channel length to main channel length (km/km). Side channels are secondary flow paths with visible surface water that were separated from the main flow path by vegetated islands and were within the functional floodplain.
Side channel node density	Number of junctions between side channels and the main channel, braids, or other side channels per kilometer of main channel. Number of nodes therefore equals two times the number of side channels.
Wood jam area	Area of wood jams per main channel length (m^2^/km). Wood jams were only digitized within the active channel and functional floodplain that had a minimum surface area of 50 m^2^

Description of habitat complexity metrics derived from aerial imagery. See Fig 2 for example of features digitized from aerial imagery.

### Productivity

Chinook salmon have complex life histories relating to variable duration of juvenile residence in freshwater prior to migration to ocean rearing habitats, where they mature before returning to freshwater as adults to spawn. Our analysis focused on subyearling Chinook salmon that migrate downstream within days to months after emerging from their redds (i.e., salmon nests) in the spring without overwintering in freshwater. Although some juvenile Chinook salmon remain in freshwater for longer periods and migrate downstream as yearlings after overwintering in freshwater rearing habitats, subyearling migrants vastly outnumber yearling migrants in Puget Sound populations [38]. Annual subyearling production was estimated for all ten watersheds from juvenile fish traps operated by Washington Department of Fish and Wildlife (WDFW), Tulalip Tribes, Stillaguamish Tribe of Indians, Puyallup Tribe of Indians, and Lummi Nation. The availability of juvenile fish trap data varies among the watersheds considered in this analysis, with trapping data available beginning in 1996–2009 (depending on the watershed) and an average of 13 years of trapping data per watershed were available through the 2015 outmigration year.

The juvenile fish trapping gear typically consists of rotary screw traps or incline-plane traps designed to sample a portion of the surface water flowing downstream in a river during the typical downstream juvenile salmon migration period (roughly January–August, depending on the watershed). These floating juvenile fish traps sample a portion of the water column and wetted width of the river at a fixed location [39]; we used mark-recapture approaches to estimate capture efficiency and hence population abundance [40]. A known number of fish were marked with dye or fin clips, and released upstream of the trapping site to estimate capture efficiency during sampling periods within a migration year using a Petersen equation as follows:

$$E_{i}=\frac{m_{i}}{M_{i}}$$ Eq 1

where

*E*_*i*_ = estimated trap efficiency during period *i*
*m*_*i*_ = number of marked fish captured during period *i*
*M*_*i*_ = number of fish marked and released during period *i*.

Total estimated abundance of downstream migrants for each period was then estimated as follows:

$$N_{i}=\frac{n_{i}}{E_{i}}$$ Eq 2

where

*N*_*i*_ = estimated number of juvenile fish migrating downstream during period *i*
*n*_*i*_ = number of fish captured at the trap during period *i*.

Following the general approach of [41], we corrected for within-season differences in capture efficiency by stratifying mark-recapture data across multiple release events, or periods, within each migration year. In some cases, these capture efficiencies are estimated through predictive models relating capture efficiency to environmental variables (river flow, turbidity) at trap sites. Capture efficiency is generally determined by the river channel configuration in the immediate vicinity of the trap site (scale < 1 km) and river conditions that often change throughout the season (flow, turbidity) and between years. Therefore, adjusting total annual catches at each trap by within year capture efficiencies are necessary to compare productivity estimates between systems at landscape scales. For a more detailed example of juvenile abundance estimations included in our analysis see [22] and [42].

Juvenile fish traps are occasionally unable to sample during the typical juvenile migration period due to high river flows, debris fowling, or mechanical problems. Linear extrapolations were used to estimate abundance during such trap outages, and in some cases, periods before and after trap deployments [22]. Outages typically lasted from a few days to a week and were often associated with high river conditions occurring earlier in the season (January—March). By using average catch before and after an outage, abundance estimates during these periods were conservative in the sense that they did not assume increased movement during higher river flows, as one might expect if increased velocity displaced fish.

Weekly expanded abundance estimates (e.g., corrected for capture efficiency) were further subdivided into fry and parr migrant types based on bimodal size and timing patterns for subyearling Chinook [22,42]. Subyearling fry are smaller (< 45 mm in length) and migrate downstream soon after emergence (days to weeks) [43–44]. Subyearling parr migrants rear longer (weeks to months) in the freshwater habitats upstream of the traps and migrate downstream later at larger sizes (≥ 45 mm in length). Given the differences in rearing duration above the traps prior to downstream migration, the distinction between fry and parr migrants from total subyearling migrants was important given the hypothesized relationships between subyearling productivity and freshwater habitat complexity. Weekly expanded estimates of total subyearling Chinook abundance, and the estimated abundance of fry and parr migrant components, were then summed for each juvenile fish trap for each of the 6–18 years of trapping data available for each watershed.

To derive annual productivity rates for subyearling Chinook for all ten watersheds, we paired the annual subyearling Chinook abundance estimates with the estimated abundance of adult Chinook salmon that contributed to each juvenile cohort. The majority of returning adults spawn upstream of the juvenile fish traps considered in this analysis. Spawner abundance estimates for each year, obtained from WDFW [45], were primarily based on redd (i.e., salmon nest) counts. These abundance estimates represent the number of Chinook salmon spawning naturally in the river, and include both hatchery-origin and natural-origin adult spawners. In the Skagit, Cedar and Dungeness rivers, virtually all spawning occurs upstream of juvenile fish traps. In the Green, Puyallup, and Nisqually rivers, some spawning occurs below the juvenile fish traps. Spawner estimates for these watersheds were adjusted based on the proportion of spawners upstream from the juvenile fish traps where sufficient distribution information were available. To calculate annual productivity rates for subyearling Chinook, we divided annual subyearling Chinook abundance estimates for each juvenile fish trap by the estimated number adult Chinook salmon that spawned naturally above each juvenile fish trap the previous fall. In this way, we derived our productivity rates as fry per spawner (FpS, total annual fry production/adult spawners), parr per spawner (PpS, total annual parr production/adult spawners), and total subyearlings (fry + parr) per spawner (SpS, total annual subyearling production/adult spawners) for each year and watershed.

Chinook salmon in Puget Sound are listed as threatened under the Endangered Species Act (ESA). All fish handling methods used to produce productivity estimates were performed under annually reviewed protocols approved by the National Marie Fisheries Service (NMFS) under the ESA 4(d) rule's research limit and the Northwest Indian Fisheries Commission (NWIFC) to minimize impacts upon individuals and the population. A mild anesthesia (Tricaine Methanesulfonate) was used to reduce juvenile handling time at all juvenile fish traps, and handling was limited by NMFS and NWIFC to annually established catch limits. Adult abundance estimates primarily employed visual methods (redd surveys via ground or aerial surveys) that did not require fish handling.

### Statistical analysis

We considered habitat complexity, peak river flows, and adult spawner densities as potential predictors of subyearling Chinook productivity. Given that collinearity among our suite of habitat complexity metrics was likely, we used Principle Components Analysis (PCA) to reduce our habitat complexity metrics to uncorrelated principle components (PC) scores for each of the ten watersheds surveyed in this study. PCA is a common data reduction solution where strongly collinear variables can be reduced to uncorrelated components that capture most of the variance that can be used as predictors in regression analyses, which are sensitive to collinearity. We used all five habitat complexity metrics derived for each of the ten watersheds (Table 1) as inputs for the PCA to derive PC scores for each of the watersheds that describe most of the variance in measured habitat complexity. We derived PCs using the prcomp function in R statistical software [46], which uses a singular value decomposition of the data matrix rather than covariance matrix eigenvalues.

We derived recurrence intervals from peak annual flows at United States Geological Survey monitoring stations located within each watershed (Fig 1). All available data through water year 2015 were used to derive recurrence intervals for each water year as;

$$T=\frac{\left(n+1\right)}{m}$$

Where *T* = recurrence interval in years, *n* = number of years of data, and *m* = rank of the peak annual river discharge in each year [47]. The number of water years available to derive recurrence intervals for each watershed ranged from 68–99 years (78 years on average).

Our analysis assumes that peak river flows typically occur in the fall and winter months in our study area, which overlaps with the incubation period (August to March) for Chinook eggs deposited by spawning adults that spawn late summer through fall. Therefore, we paired annual productivity rates with flow recurrence intervals for the water year following the calendar year in which eggs were deposited to account for peak flow conditions during the incubation period of each cohort.

Annual densities of spawning Chinook adults (spawners/km) were derived from the same annual adult spawner abundance estimates that were used to calculate annual productivity rates, with annual abundance being divided by the total length (km) of channel habitat (main, braid, and side channel) mapped in this analysis that fell within WDFW published spawner distributions for all Chinook run types within each watershed [48]. These annual spawner densities were paired with the annual productivity estimates for the subsequent calendar year to represent the density of adult Chinook salmon that produced each cohort of migrating subyearling Chinook salmon.

We developed separate linear mixed-effects regression models for the 6–18 years of annual FpS, PpS, and SpS productivity rates derived for each watershed. Habitat complexity (PC1 and PC2), peak flow recurrence interval, spawner density, and year (representing the broodyear or year in which the eggs were deposited) were considered as predictors for all three linear mixed-effects regression models. We log transformed the dependent variables, (expressed as logSpS, logFpS, and logPpS) due to geometric variation typical of recruitment rates in fish populations. Spawner densities and peak flow recurrence intervals were also log transformed (expressed as logSD and logRI) to improve normality of the predictor variables.

All factors were fixed effects except year, which was considered a random effect given that we were not interested in describing differences between years. We used a full subsets regression approach with all possible subsets and a maximum of two factors with predictors being standardized (mean of 0 and standard deviation of 1). Predictors included in the selected models were evaluated for collinearities using Variance Inflation Factor (VIF) tests, with the assumption that VIF values over 3–4 indicate severe collinearities that could adversely influence model selection and interpretation [49]. Models were developed and tested using the lme4 [50], Multi-Model Inference [51], and CAR [52] packages in R statistical software [46]. We selected models based on Akaike’s Information Criterion adjusted for small sample sizes, with ΔAICc scores of 0–7 being considered plausible models [53–54].

## Results

### Habitat complexity

We found habitat quantity and complexity variations among watersheds with 3–11 fold differences in complexity between basins (S1 Table), and significant correlations between some complexity metrics (Pearson’s correlation: *p* < 0.05). Wood jam densities were positively correlated with side to main channel length ratios (Pearson’s *r* = 0.75), braid to main channel length ratios (Pearson’s *r* = 0.76), and braid channel node densities (Pearson’s *r* = 0.65). The ratios of side and braid channel to main channel lengths were also positively correlated with their respective node densities (Pearson’s *r* = 0.70 and 0.91, respectively).

Principle Components Analysis (PCA) indicated that 91% of the variance in habitat complexity could be explained by the first two principle components (Table 2). The first principle component (PC1) explained 56% of the variance and the second principle component (PC2) explained an additional 35% (Table 2). Braid and wood jam metrics (braid to main channel length ratio, braid node density, and wood jam density) collectively contributed 72% to PC1 loadings, with side channel metrics (side to main channel length ratio and side channel node density) contributing the remaining 18% (Table 2). In contrast, side channel metrics contributed 59% to PC2 loadings, with braid channel metrics contributing an additional 38% and wood jam density contributing 4% (Table 2).

**Table 2:** Principle component loadings of habitat complexity metrics.

Metric	PC1	PC2	PC3	PC4	PC5
side: main channel length ratio	0.399 (*19%*)	0.523 (*26%*)	-0.407 (*20%*)	-0.510 (*29%*)	-0.377 (*19%*)
braid: main channel length ratio	0.498 (*23%*)	-0.406 (*20%*)	-0.059 (*3%*)	-0.441 (*25%*)	0.624 (*31%*)
side channel node density	0.188 (*9%*)	0.655 (*33%*)	0.631 (*32%*)	0.044 (*3%*)	0.368 (*18%*)
braid node density	0.481 (*23%*)	-0.356 (*18%*)	0.561 (*28%*)	-0.014 (*1%*)	-0.572 (*28%*)
wood jam density	0.571 (*27%*)	0.073 (*4%*)	-0.344 (*17%*)	0.737 (*42%*)	0.080 (*4%*)
Standard deviation	1.680	1.316	0.622	0.209	0.128
Proportion of Variance	0.564	0.346	0.077	0.009	0.003
Cumulative Proportion	0.564	0.911	0.988	0.997	1.000

Principle component (PC) loadings from principle components analysis of habitat complexity metrics, with percent contributions of each metric to the loadings for each PC included in parenthesis.

**Fig 3:**
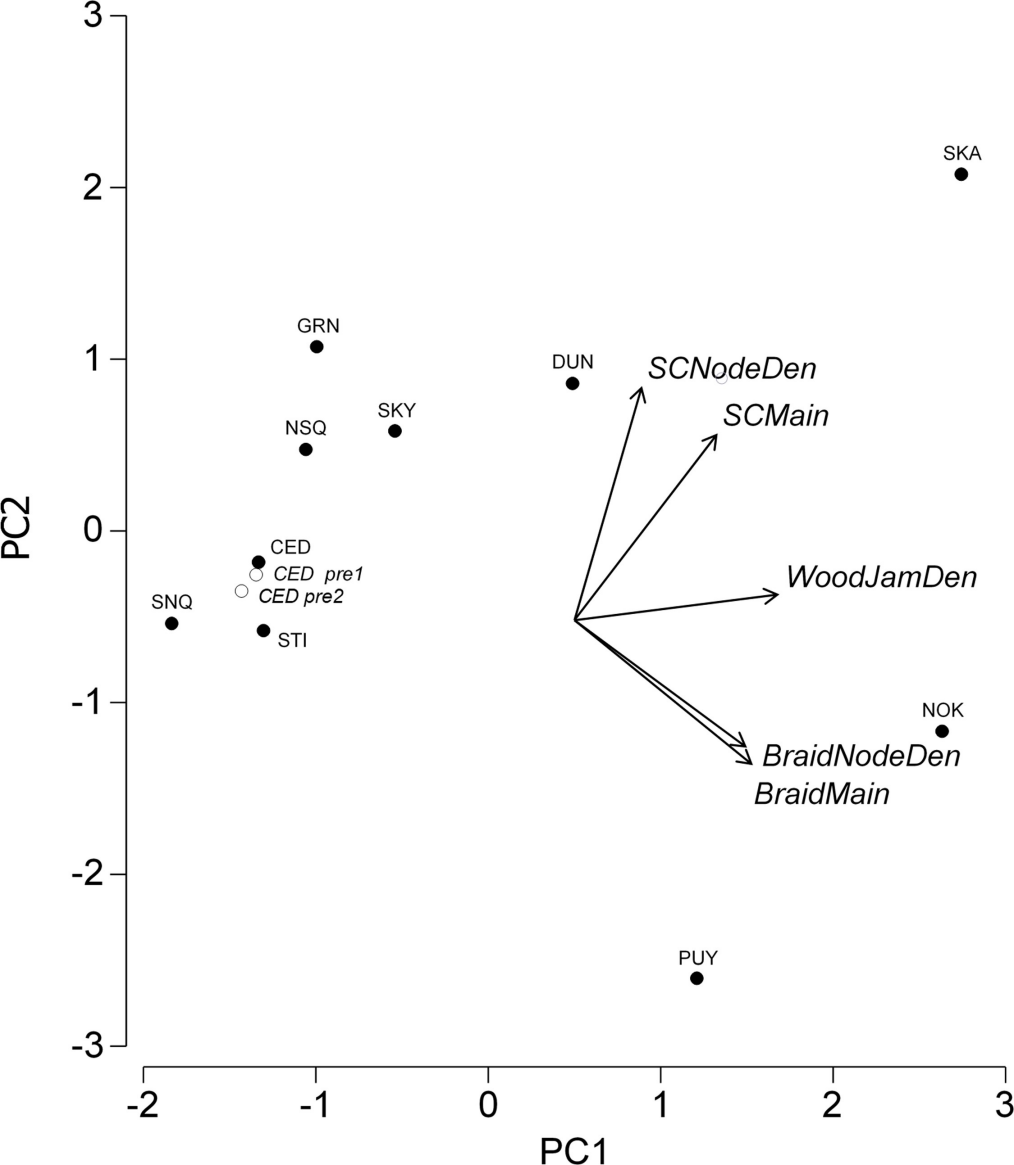
Principle components analysis (PCA) bi-plot for watershed habitat complexity metrics. PCA scores for the first two principle components (PC1 and PC2) of habitat metrics for all watersheds (PUY = Puyallup, NOK = Nooksack, CED = Cedar, SNQ = Snoqualmie, STI + Stillaguamish, NSQ = Nisqually, SKY = Skykomish, DUN = Dungeness, GRN = Green, and SKA = Skagit). The PC scores for each watershed are shown as solid black circles, with open circles showing PC scores for the Cedar River with a one side channel construction project removed (*CED pre1*) and an additional side channel construction project removed (*CED pre2*). Vectors show loadings for each metric, with labels for each metric that include side channel to main channel (*SCMain*) and braid channel to main channel (*BRMain*) ratios; the density of side channel nodes (*SCNodeDen*) and braid channel nodes (*BRNodeDen*); wood jam density (*WoodJamDen*).

Ordination of PC1 and PC2 scores show basin scale habitat complexity gradients that were not correlated with drainage areas above smolt traps (Pearson’s *r* = 0.25 (*p* = 0.485) for PC1, and -0.07 (*p* = 0.838) for PC2). We observed increasing habitat complexity along the PC1 axis that represented increasing wood jam densities from the Snoqualmie to Skagit River (Fig 3 and S1 Table). Along the PC2 axis, watersheds scored along a gradient of increasing habitat complexity from the Puyallup to Skagit River (Fig 3). Side channel metrics were the largest contributors to PC2 loadings (Table 2), and the Puyallup River had the lowest side length ratio and node density (S1 Table). In contrast, Skagit River had the highest side channel length ratio and node density (Fig 3 and S1 Table). In the Cedar River restoration example, we attributed 1,276 m of side channel habitat to two projects that constructed a total of three side channels [55–56]. These constructed habitats increased Cedar River PC1 scores from -1.40 to -1.31 and PC2 from -0.35 to -0.17 (Fig 3).

### Juvenile productivity

Skagit and Cedar River had the highest average subyearling Chinook per spawner (SpS) rates (315 and 326 SpS, respectively), while Snoqualmie and Puyallup River had the lowest (65 and 68 SpS, respectively) (S2 Table). Annual fry per spawner (FpS) production rates were higher than parr per spawner (PpS) in most years in Skagit, Cedar, Green, and Skykomish Rivers (S2 Table). In contrast, PpS rates were higher than FpS in most years within the Dungeness, Snoqualmie, and Stillaguamish Rivers (S2 Table). Mean annual capture efficiency estimates at each juvenile fish trap were not significantly correlated with annual productivity estimates for SpS, FpS, or PpS (Pearson’s correlation: *p* > 0.05).

**Fig 4:**
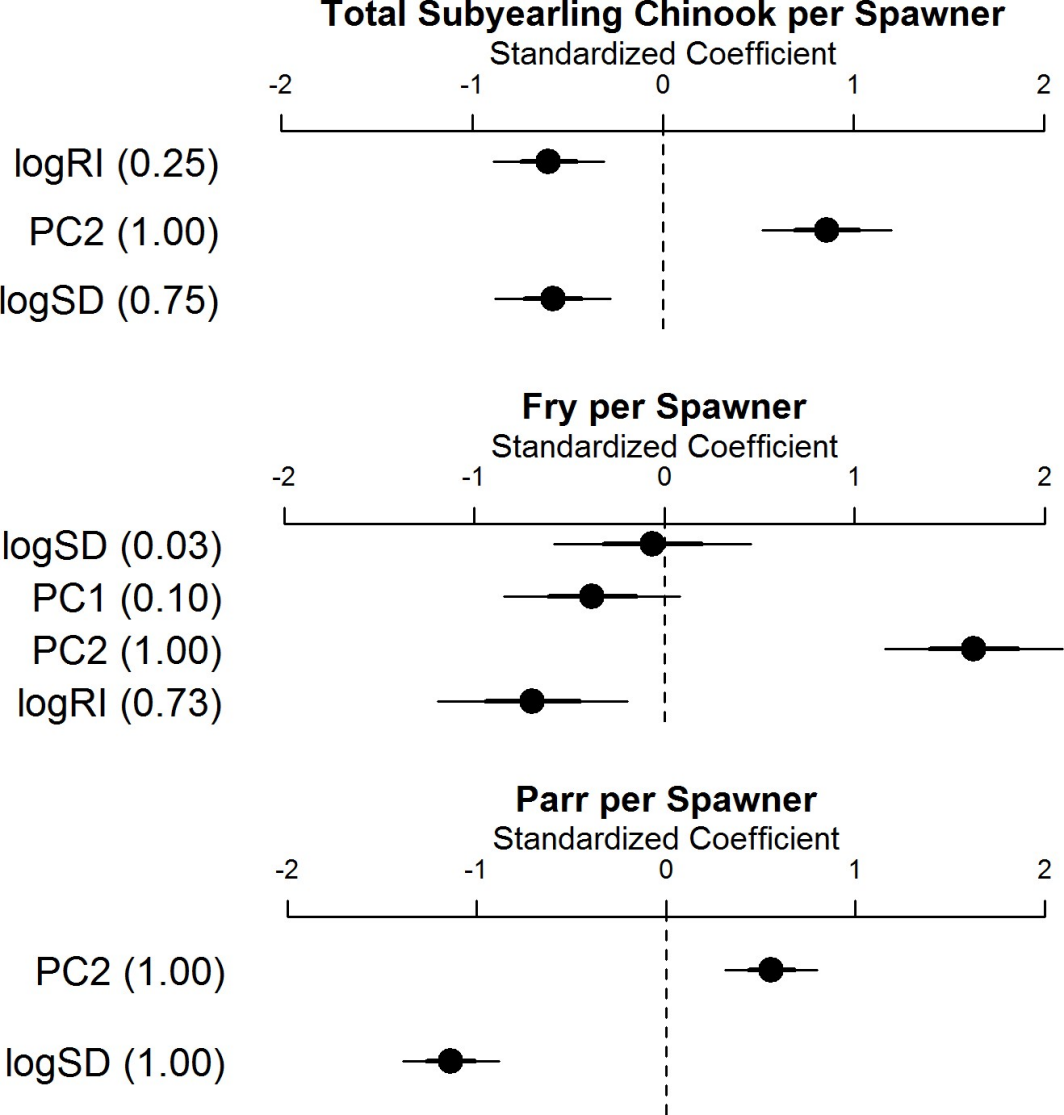
Standardized coefficient plots of AICc selected models. Standardized coefficient plots for log transformed total subyearling Chinook per spawner, fry per spawner, and parr per spawner models considering principle components for habitat complexity (PC1 and PC2), log transformed peak flow recurrence interval (logRI), and log transformed spawner density (logSD). Points represent the standardized coefficient estimate for each factor from the averaged model or top model if only one model was selected. Thick bars represent one standard deviation and a 68% confidence interval for the standardized coefficient, while the thin bar represents two standard deviations and a 95% confidence interval. The importance for each factor are shown in parenthesis following each factor, which is derived from the sum of AIC weights for models that include the factor with a value of 1.00 indicating that the parameter was included in all selected models.

**Fig 5:**
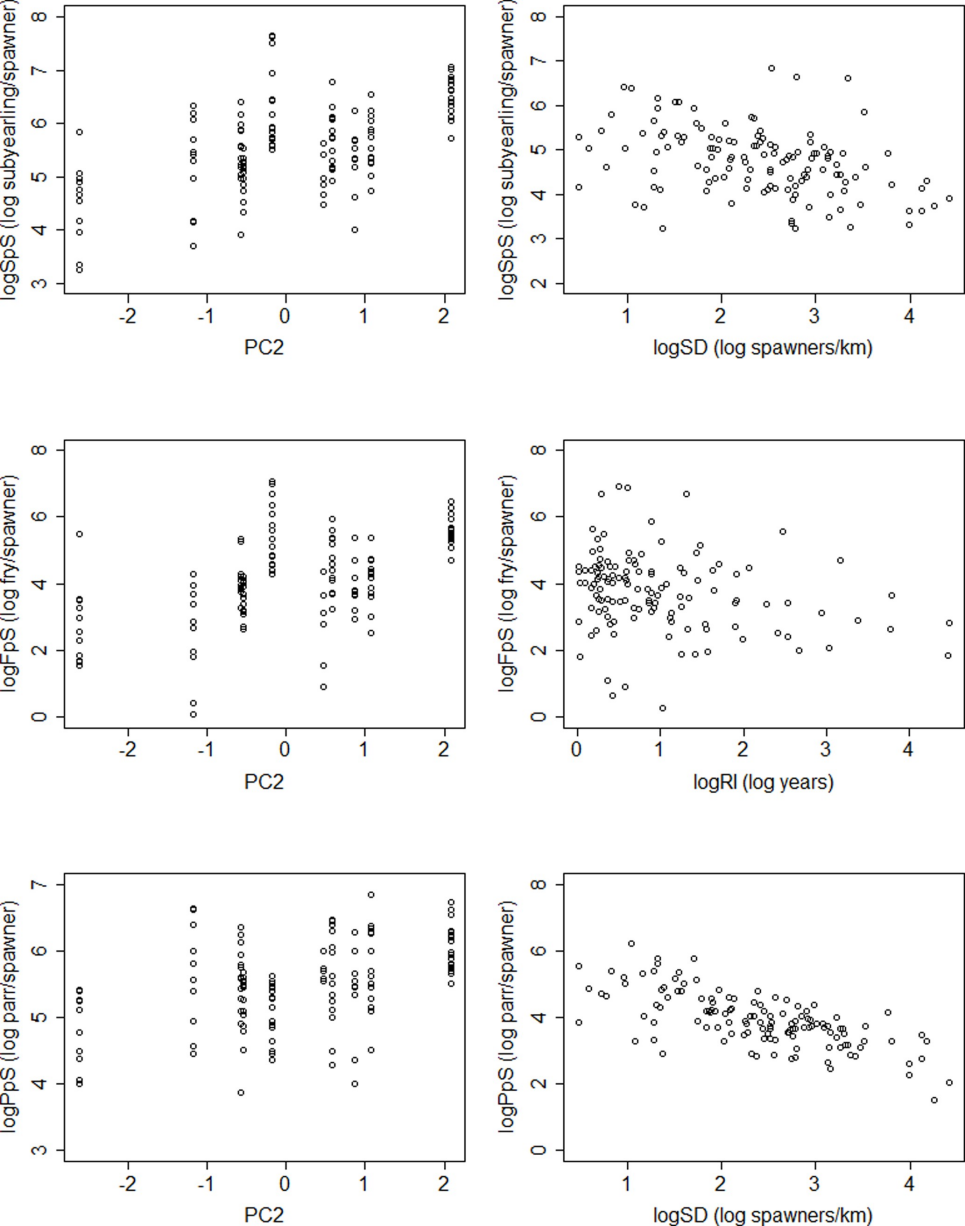
Partial effects plots showing predicted productivity rates. Partial effects plots of log transformed total subyearling Chinook per spawner (logSpS, top plots), fry per spawner (logFpS, middle plots), and parr per spawner (logPpS, bottom plots) from the top models (Table 3). The left panels show predicted productivity with habitat complexity (PC2) while controlling for broodyear and log transformed spawner density (logSD) for logSpS and logPpS, and logFpS while controlling for broodyear and log transformed recurrence interval (logRI). The right panels show predicted productivity with log transformed spawner density for logSpS and logPpS, and with recurrence interval for logFpS while controlling for broodyear and habitat complexity (PC2).

Among the models that were selected (ΔAICc < 7), collinearity tests between variables included in the selected models indicate that Variance Inflation Factors (VIF) ranged from 1.00–1.15. Habitat complexity described by PC2 was included in all selected models for log transformed subyearling Chinook per spawner (logSpS), fry per spawner (logFpS), and parr per spawner (logPpS) productivity rates (Table 3). Models of logSpS that paired PC2 with either log transformed spawner density (logSD) or log transformed peak flow recurrence interval (logRI) exhibited the strongest support. For logFpS, four models included just PC2, and models that paired PC2 with either logRI, logSD, or PC1 were well-supported. For logPpS, we found strong support for a single model that paired PC2 with logSD. In all selected models, PC2 was positively related to productivity whereas logSD and logRI were negatively related to productivity (Figs 4 and 5). PC2 had a greater influence on logFpS than either logSpS and logPpS as evidenced by standardized coefficients that were approximately two and three times larger in the selected models, respectively (Fig 4). LogRI had similar influence on logSpS and logFpS, but the influence of logRI was less than that of PC2, whereas logSD had approximately two times the influence on logPpS compared to PC2 (Fig 4).

**Table 3:** Full subsets linear mixed effects regression model statistics for subyearling Chinook productivity rate models.

Model	Parameters*	adj R^2^	df	logLik	AICc	ΔAICc	weight
logSpS	PC2, logSD	0.31	5	-149.96	310.4	0.0	0.74
	PC2, logRI	0.30	5	-151.05	312.6	2.2	0.25
	PC2	0.22	4	-156.12	320.6	10.2	0.01
	PC2, PC1	0.24	5	-155.78	322.1	11.6	0.00
	logRI	0.14	4	-161.82	332.0	21.5	0.00
	logRI, PC1	0.17	5	-160.78	332.1	21.7	0.00
	logRI, logSD	0.15	5	-161.92	334.3	23.9	0.00
	PC1	0.08	4	-165.42	339.2	28.8	0.00
	logSD	0.06	4	-166.60	341.5	31.1	0.00
	logSD, PC1	0.09	5	-165.71	341.9	31.5	0.00
logFpS	PC2, logRI	0.35	5	-214.15	438.8	0.0	0.73
	PC2	0.31	4	-216.93	442.2	3.4	0.13
	PC2, PC1	0.32	5	-216.11	442.7	3.9	0.10
	PC2, logSD	0.31	5	-217.35	445.2	6.4	0.03
	logRI, logSD	0.11	5	-232.67	475.8	37.1	0.00
	logRI	0.07	4	-234.71	477.7	39.0	0.00
	logRI, PC1	0.08	5	-234.86	480.2	41.4	0.00
	logSD	0.05	4	-236.00	480.3	41.5	0.00
	logSD, PC1	0.05	5	-236.37	483.2	44.4	0.00
	PC1	0.02	4	-237.87	484.1	45.3	0.00
logPpS	PC2, logSD	0.44	5	-132.90	276.3	0.0	0.98
	logSD, logRI	0.40	5	-137.19	284.9	8.6	0.01
	logSD, PC1	0.38	5	-138.73	288.0	11.7	0.00
	logSD	0.34	4	-141.47	291.3	15.0	0.00
	logRI, PC1	0.21	5	-152.56	315.6	39.3	0.00
	PC1	0.13	4	-156.62	321.6	45.3	0.00
	logRI	0.13	4	-157.19	322.7	46.4	0.00
	PC2, PC1	0.14	5	-157.28	325.0	48.7	0.00
	PC2, logRI	0.14	5	-157.44	325.4	49.1	0.00
	PC2	0.05	4	-161.68	331.7	55.4	0.00

Full subsets linear mixed effects regression model statistics for log transformed subyearling Chinook per spawner (logSpS), fry per spawner (logFpS) and parr per spawner (logPpS) rate models.

*PC1 and PC2 are the first two principle component scores from the habitat complexity principle component anlaysis; logSD is log transformed spawner density; and logRI is log transformed peak flow recurrence interval.

**Fig 6:**
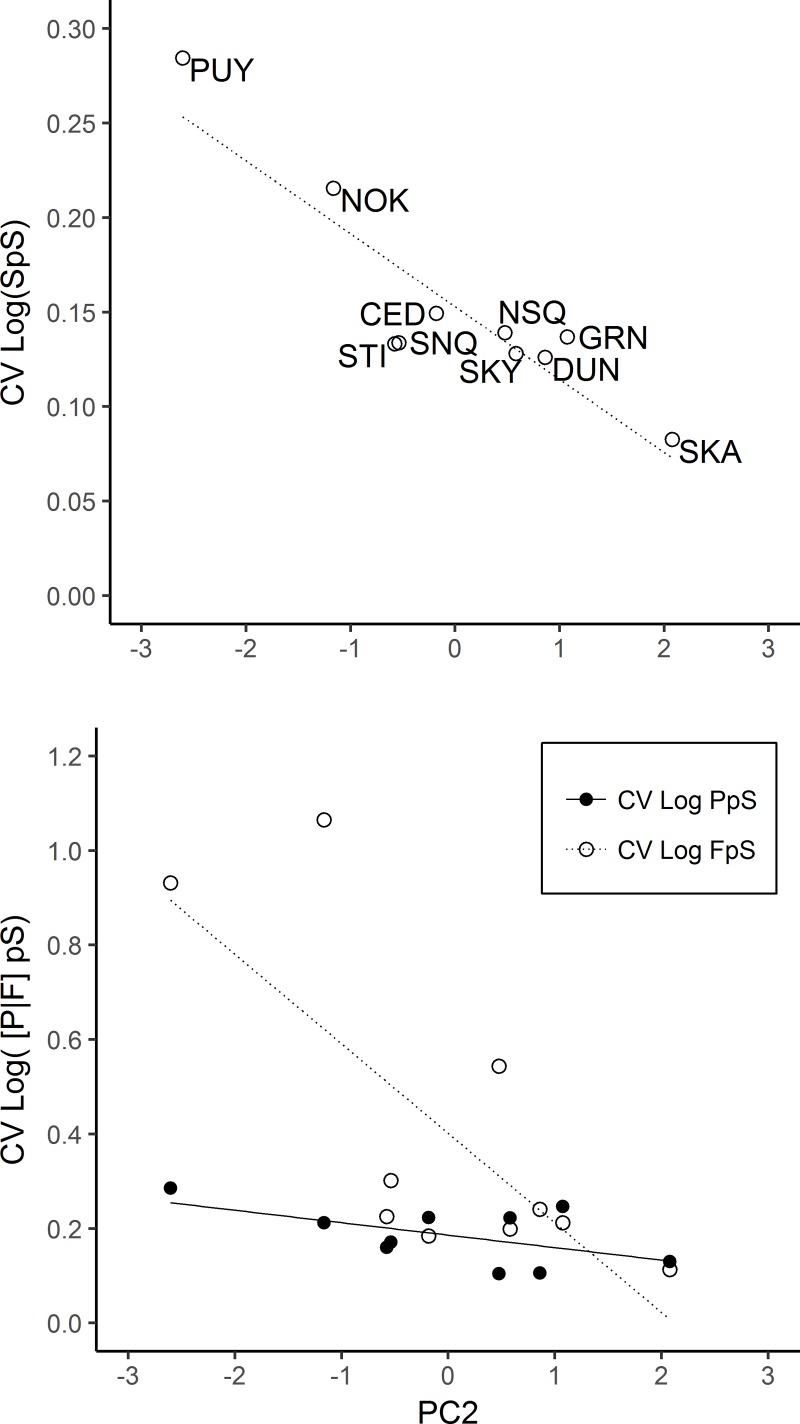
Relationship between the coefficient of variation and productivity. Coefficient of variation in log transformed total subyearling Chinook per spawner (CV logSpS) and habitat complexity (PC2) (top), and the coefficient and variation in log transformed fry per spawner and parr per spawner (CV logFpS and CV logPpS, respectively) and habitat complexity (PC2) (bottom). Linear regression trend lines are shown in each plot for CV logSpS, CV logFpS, and CV logPpS. Watersheds in increasing order of PC2 scores shown in both plots are Puyallup, Nooksack, Stillaguamish, Snoqualmie, Cedar, Nisqually, Skykomish, Dungeness, Green, and Skagit.

We also observed relationships between annual variation in productivity and habitat complexity (Fig 6). The coefficient of variation (CV) in logSpS and logFpS within each watershed decreased with increasing PC2 scores, from the Puyallup to the Skagit River (Fig 6). The relationship between PC2 and the CV of logSpS (linear regression: F-statistic = 3.5, df = 1,8, adjusted R^2^ = 0.78, *p* < 0.001) and logFpS were statistically significant (linear regression: F-statistic = 9.8, df = 1,8, adjusted R^2^ = 0.49, *p* = 0.014), whereas we did not detect a significant relationship between the CV of logPpS and PC2 (linear regression: F-statistic = 3.7, df = 1,8, adjusted R^2^ = 0.23, *p* = 0.091) (Fig 6).

## Discussion

### Habitat complexity

We quantified habitat complexity differences at basin scales for ten Puget Sound basins using census-based, large-scale, remotely-sensed salmon habitat-monitoring metrics derived from readily available aerial imagery [28]. This approach revealed 3–11 fold differences in complexity between basins that highlight potentially important differences in geomorphology, hydrology, or land use among Puget Sound’s large rivers. Furthermore, the calculation of each habitat complexity metric corrects for main channel length, thereby standardizing habitat quantity metrics across watersheds. Hence, Skagit River is Puget Sound’s largest watershed and consistently scored highest in habitat quantity for all metrics, but the Puyallup and Nooksack Rivers scored highest for braid channel length ratios and node densities (S1 Table). Therefore, our method better highlights potentially important differences in geomorphology, hydrology, or land use among Puget Sound’s watersheds independent of their size.

Our analysis also revealed that several of our habitat complexity metrics had strong positive correlations (Pearson’s *r* = 0.65–0.91), which were somewhat expected. For example, wood jam densities were positively correlated with the ratio of side and braid channels to main channel length (r = 0.75 and 0.76, respectively). This correlation could be related to large woody debris accumulations initiating hydrological processes that form and maintain side and braid channels, or the presence of geomorphology attributes (e.g., low gradient wide valley bottoms) that are conducive to both wood accumulation and side channel formation [32]. However, we also observed departures from these correlative patterns in watersheds, such as the Skykomish River, where higher than average side to main channel ratios were associated with lower than average wood jam densities. By using principle components analysis (PCA), we reduced our suite of habitat complexity metrics to uncorrelated principle components (PC) that explained most of this potentially important variance in watershed-scale habitat complexity. Our basin PC scores were not correlated with habitat quantity and therefore represented habitat complexity that was independent of basin size.

Furthermore, we were able to demonstrate that habitat associated with restoration projects in the Cedar River watershed resulted in measurable changes in both habitat complexity and PC scores from our census-based remote sensing approach (Fig 3). In addition to capturing habitat complexity differences and restoration related changes, we anticipate that our approach will also detect changes over time associated with natural processes, land use changes, or other hydromodifications (e.g., channel straightening, confinement, and disconnection of floodplain habitats). Given that regional-scale aerial imagery is readily available and frequently updated (e.g., 1–3 years), our census-based remote sensing approach is likely amenable to tracking changes in the condition of large river systems over time.

### Habitat and productivity

Our model selection approach revealed that habitat complexity as measured by PC2 was a strong predictor of subyearling Chinook salmon productivity. All selected models included PC2 (Table 3), which primarily describes the density of side and braid channel connections and length relative to the length of main channel within a watershed. Log transformed total subyearling per spawner (logSpS), fry per spawner (logFpS), and parr per spawner (logPpS) productivity rates were positively related to habitat complexity as described by PC2 (Figs 4 and 5). Given that PC2 did not correlate with basin size, our results indicate habitat complexity, as described by PC2, and productivity were not merely functions of watershed size. Although these relationships between habitat complexity and subyearling productivity are correlative, our findings agree with studies showing how habitat complexity might increase salmon productivity. For example, other measures of habitat complexity have been positively related to demographics like fish size, condition (e.g., relationship between weight and length, lipid content, etc.), behavior, and survival [11–12]. Therefore, we postulate that gradients in our basin-scale habitat complexity metrics captured coarse differences in habitat structure that related to aggregate subyearling Chinook productivity among Puget Sound’s large rivers. This was encouraging given that Puget Sound’s basins represent a range of hydrological characteristics, from rainfall-dominated to snowmelt-dominated watersheds [21], and a variety of geomorphological settings and land use patterns [57–58].

Interestingly, our model selection results suggested that habitat complexity described by PC1 was not a strong predictor of subyearling Chinook productivity compared to PC2. Looking at the differences in loadings for the first two principle components, we see that PC1 scores were more influenced by wood jam densities and braid channel metrics (density of braid nodes and length relative to main channel length) compared to PC2 scores. Loadings for PC2 scores had very little influence from wood jam densities and were mostly driven by side channel metrics (density of side nodes and length relative to main channel length). Although numerous studies have demonstrated positive associations between juvenile salmon and the habitats associated with large woody debris accumulations [23,33], the mosaic of habitats and slower water migration pathways created by bifurcations of the main channel in watersheds with higher densities of side channels may offer more direct benefits to subyearling Chinook incubation survival and rearing habitat [30]. Side channels serve as rearing habitat for the subyearling parr life history type; indeed, higher densities of subyearling Chinook salmon have been observed in Skagit River side channel habitats compared to accumulations of large woody debris [59]. Furthermore, side channels likely help disperse the energy of winter high flow events that would otherwise be contained by the main channel in reaches that lack side channels, and this likely confers benefits to survival during the egg incubation phase [22].

Our model selection approach also indicated that logSpS and logFpS were best described by models that included log transformed peak flow recurrence interval (logRI) with habitat complexity described by PC2 (Table 3). LogRI was a strong predictor of logSpS and logFpS and was negatively related productivity (Figs 4 and 5). This agrees with studies demonstrating that high flows during the period in which eggs are incubating in riverine substrates can reduce survival. For example, moderate and large flow events can induce substrate movement and scour that displaces eggs and embryos, or causes eggs to become buried from sediment deposition [21–22,42,60–61]. Given numerous examples demonstrating strong negative effects of peak flows on juvenile salmon productivity, we were surprised to find that habitat complexity described by PC2 was a stronger predictor of logSpS and logFpS than logRI (Fig 4).

Model selection also showed that logSpS and logPpS were best described by models that included log transformed spawner density (logSD) in combination with habitat complexity (Table 3). LogSD was negatively related to productivity rates (Figs 4 and 5) as was previously found in the Skagit and Green rivers, where abundance of parr that rear longer in freshwater habitats was best explained by density-dependent models [22,42]. Density-dependent effects, whereby increasing egg deposition or spawner abundance do not provide commensurate increases in parr abundance, are likely related to limitations on juvenile rearing habitat, leading to earlier downstream movements of juveniles as fry migrants. These fry movements might be voluntary, if fish actively seek unoccupied rearing habitats, or involuntary, if fish failing to acquire rearing habitats providing velocity refuge are swept downstream during high flow events [22,42]. Density-dependent effects might also be related to survival, if parr rearing in freshwater suffer higher mortality in years of greater adult abundance due to resource limitations. In contrast to the selected logSpS and logFpS models, logSD was a stronger predictor of logPpS than habitat complexity (Fig 4), although the only selected logPpS model included habitat complexity (Table 3).

Considering the Cedar River restoration example, we predicted subyearling Chinook productivity with our selected models based on habitat measures before and after two restoration projects. The addition of side channel habitat associated with these projects was detectable using our remote sensing approach and these projects increased PC2 scores for the Cedar River watershed. Using our selected models, increased PC2 scores associated with the restoration projects increased predicted fry productivity by 0.4–1.8% and parr productivity by 1.7–17.9% under the range of observed spawner densities and peak flow recurrence intervals in Cedar River. The logFpS model showed that the increases in predicted FpS associated with the post-restoration increase in PC2 score was equivalent to a reduction in peak flow recurrence interval from a 13.5 year event to a 10-year event. The logPpS model indicates that the increased PC2 score associated with post-restoration conditions was equivalent to reducing spawner densities in the Cedar River by 4.4%. These examples demonstrate how modest increases in habitat complexity through restoration may increase productivity of subyearling Chinook in Puget Sound rivers.

If habitat complexity does in fact buffer subyearling Chinook productivity from factors like spawner density and peak flows as our results suggest, we would expect an inverse relationship between complexity and variation in productivity. Our results support this hypothesis given that the coefficient of variation (CV) in logSpS and logFpS had statistically significant negative relationships with PC2 (Fig 6). Interestingly, the relationship between CV in logPpS and PC2 was not statistically significant. However, our model selection results revealed that logSD was a better predictor of logPpS compared to PC2 and therefore variations in logPpS may be more closely related to logSD. The significant relationships between PC2 and the CV of logSpS and logFpS suggests that watersheds with greater complexity offer greater population resilience with more consistent rates of productivity in the face of environmental variation. Basin size was not related to productivity rates or CV for productivity, thus suggesting watersheds with greater complexity and not just more habitat offer greater resilience to their fish populations. If greater habitat complexity does increase population resilience by reducing variation in productivity rates, restoring floodplain habitat complexity may also offset climate change impacts. For example, snowmelt dominated watersheds like Skagit River may transition to rainfall dominated hydrographs, which could increase peak flows during juvenile salmon incubation periods up to 32% by the 2080s [62–63]. The Cedar River restoration example shown here, and the relationship between productivity variation and habitat complexity, show how increased habitat complexity through restoration may serve as a way to dampen climate change impacts to salmon recovery [24].

### Conclusions

The results of our analysis showcase that large river habitat complexity is measurable, varies among large river watersheds, and is a strong correlate of subyearling Chinook productivity for Puget Sound populations. Furthermore, we demonstrated that our approach detected changes in habitat complexity associated with actual restoration projects. We also hypothesize that our approach is scale-able and can be applied in other watersheds where population recovery and conservation of salmonids or other aquatic species could benefit from consistent habitat status and trends monitoring. Hence, our approach is likely amenable to indicator-based approaches to tracking changes in the condition of freshwater ecosystems over time and inform salmon recovery evaluation at regional and watershed scales [28]. We expect that broad episodic natural events and large-scale restoration efforts such as dam removal, levee set backs, and channel reconnections can be detected by our approach, as well as smaller scale changes at longer time scales [57].

More broadly, we expect that metrics describing status and trends in habitat complexity will be useful for tracking landscape effects upon the demography of populations. In the aquatic realm, these include headwater streams [4,15,64] and estuarine nursery areas [65–67]. In terrestrial systems, metrics of land use change [57] and forest structure [1] are likely to exhibit long-term changes over time and space and have strong demographic impacts. Determining scalable metrics that detect changes in landscape features will likely shed light on far-reaching impacts such as urbanization and climate change upon population responses in many other species.

## Supporting information

S1 Table Habitat quantity and complexity metrics. Quantity and complexity metrics were derived from aerial imagery analysis within the survey extents for each Puget Sound watershed as shown in Fig 1. These metrics are shown in the digitized coordinate reference system (WGS 84 Pseudo Mercator) and local coordinate reference system (WGS 84 UTM Zone 10N).
(XLSX)

S2 Table Productivity rates for subyearling Chinook salmon. Puget Sound subyearling Chinook productivity rates are expressed as total subyearling per spawner (SpS), fry per spawner (FpS), and parr per spawner rates (PpS). See Fig 1 for map of large river watersheds in Puget Sound.
(XLSX)
